# Barriers Limiting Equity in Access to Pretransplant Evaluation Among Predialysis Patients: A Cohort Study

**DOI:** 10.1097/TXD.0000000000001872

**Published:** 2025-10-20

**Authors:** Julie Piotte, Véronique Beaulieu, Sacha A. De Serres

**Affiliations:** 1 Division of Nephrology, University Health Center (CHU) of Quebec—Laval University, Quebec, QC, Canada.; 2 Division of Nephrology, Regional Affiliated University Health Center (CHAUR)—Université de Montréal, Trois-Rivières, QC, Canada.

## Abstract

**Background.:**

Kidney transplantation is the optimal treatment for end-stage renal disease; however, referral for pretransplant evaluation remains inconsistent. We aimed to compare patients deemed eligible for pretransplant evaluation with those whose eligibility status was undetermined.

**Methods.:**

We conducted a single-center retrospective cohort study of patients aged ≤80 y, followed-up in a chronic kidney disease (CKD) clinic as of June 2021. We compared demographic, clinical characteristics, and survival prognosis scores between these groups.

**Results.:**

Of the 572 patients followed in the clinic, 395 were aged ≤80 y. Among them, 203 had progressive disease, defined as CKD stage V or CKD stage IV with an estimated glomerular filtration rate (eGFR) projected to fall below 15 mL/min/1.73 m^2^ within 5 y. These 203 patients were classified by the CKD clinic medical team as follows: 46 (23%) were considered eligible for pretransplant evaluation, 78 (38%) were deemed unsuitable, and 79 (39%) had an undetermined status. There were no significant differences in sex, body mass index, ethnicity, or distance from the transplant center between eligible and undetermined patients. However, undetermined patients were older (66 ± 11 versus 58 ± 13 y, *P* < 0.01), had a higher eGFR (15 ± 5 versus 12 ± 4 mL/min/1.73 m^2^, *P* < 0.01), a greater number of cardiovascular and metabolic comorbidities (2.4 ± 1.6 versus 1.6 ± 1.3, *P* < 0.01), and a higher 3-y mortality risk score (5.9 ± 3.7 versus 3.2 ± 3.0, *P* < 0.01). Nonetheless, 43 (54%) of 79 undetermined patients were in the acceptable prognosis risk category for kidney transplantation, with a predicted 3-y dialysis mortality of ≤30%.

**Conclusions.:**

A substantial proportion of patients theoretically eligible for pretransplant evaluation had an undetermined referral status. Our findings suggest that several of these patients share similar age, comorbidity profiles, and survival prospects with those already deemed eligible for pretransplant evaluation.

## INTRODUCTION

Chronic kidney disease (CKD) is a major global health issue, affecting 9%–13% of the world’s population, including 42 million individuals in North America.^1-3^ In Canada, >50 000 patients are living with end-stage kidney disease (ESRD), of whom only 38% have a functioning kidney graft.^4^ Most patients who progress to ESRD require renal replacement therapy (RRT), with kidney transplantation being the gold standard for this population.^5^ In addition to superior outcomes, kidney transplantation is highly cost-effective, saving up to $75 000 per patient annually.^6-9^ Furthermore, preemptive kidney transplantation offers additional benefits in terms of improved patient and graft survival.^10-12^ Despite these advantages, only a minority of patients are waitlisted preemptively.^13,14^

Many barriers to transplantation, especially to preemptive transplantation, have been identified in the dialysis population, including delays in referral, socioeconomic barriers, and racial disparities.^15-17^ A large US study involving 690 dialysis facilities found that patients referred for kidney transplant evaluation, within 1 y of RRT initiation, were generally younger (54 versus 59 y old), had fewer comorbidities, and more likely of Black descent; however this last group had a lower evaluation start rate.^13^ In a different setting, a study of patients initiating RRT in France highlighted age, comorbidities, and employment status as factors limiting referral to transplantation.^18^

One potential, underappreciated issue is the heterogeneity in the referral of patients with CKD to pretransplant evaluation. Referral rates for pretransplant evaluation vary widely worldwide, with 17%–43% of patients with ESRD being referred within 1 y of dialysis initiation.^19-21^ Data on referral rates before the initiation of dialysis are even more limited, with rates ranging from just 3% to 10%.^20,22^ Although some patients are undeniably unsuitable candidates for transplant, the wide variation in referral rates suggests that subjective factors may influence the assessment of pretransplant eligibility by the CKD clinic or dialysis team. This is a crucial step in the access to transplantation because in most centers, these teams are responsible for deciding whether or not to prepare and submit a pretransplant request to the transplant team for formal evaluation.

Preemptive transplantation opportunities are particularly relevant for elderly candidates, for whom any delay could jeopardize eligibility. These patients can benefit from transplants even from older donors, who are typically excluded from standard kidney allocation. The Eurotransplant Senior Program, which matches kidneys from cadaveric donors >65 y of age with recipients of the same age group, has demonstrated reduced cold ischemia time and shorter transplant waiting times compared with the standard European allocation system.^23-25^ Additionally, improved 1- and 5-y survival rates have been observed in elderly transplant recipients compared with their counterparts on dialysis.^26,27^

Most studies on pretransplant referrals have focused on relatively young populations. Considering that Canada has a significantly aging population,^28^ we hypothesized that age might play a more prominent role in limiting pretransplant evaluation in our predialysis patients. Thus, this study aimed to characterize the differences between patients in whom a pretransplant evaluation was initiated versus those in whom it was not, within a predialysis CKD clinic setting.

## MATERIALS AND METHODS

### Study Design, Population, and Data

This is a single-center, retrospective, cohort study of CKD patients followed at a tertiary nephrology center in Quebec City, QC, which serves a population of 2 million individuals. The cohort included all patients registered in the predialysis CKD clinic as of June 1, 2021. Patients older than 80 y were excluded, as they are generally considered unsuitable for transplant by most centers.^29-31^

### Data Collection and Prognosis Score

Electronic medical records and patient charts were reviewed to collect demographic data, kidney function parameters, comorbidities, and pretransplant evaluation referral status. Data collection covered the period from the time patients were registered at the clinic through the end of December 2023. Baseline kidney function was assessed using the estimated glomerular filtration rate (eGFR), calculated with the CKD-EPI formula based on the most recent outpatient serum creatinine measurement.^32^ The eGFR slope was calculated from longitudinal serum creatinine measurements obtained during CKD clinic follow-up. Patients were prospectively followed at the clinic and deceased status was verified for each participant.

Relevant comorbidities were recorded based on the components of the prognosis score described by Dusseux et al.^33^ This screening tool, developed and validated from a national French registry of >15 000 patients, predicts the 3-y mortality risk for dialysis patients >70 y. The score was designed to assess potential transplant candidates in this population by predicting overall mortality from the first day of dialysis. The relevant comorbidities described by Dusseux et al., included in the risk score, are the following: body mass index, ischemic heart disease, peripheral vascular disease, heat failure, dysrhythmia, chronic respiratory disease, cerebrovascular disease, active malignancy, severe behavioral disorders, mobility issues, and catheter use as dialysis access at initiation. A score of <7 predicts a validated 30% 3-y mortality, a score between 7 and 9 predicts a 47% mortality, and a score >9 suggests a mortality rate >60%.^33^

### Definition of CKD Progression to ESRD

Patients were categorized as having progressive or nonprogressive CKD based on the following criteria: patients were considered “progressive” (hereafter referred to as “progressives”) if, as of June 2021, they had reached CKD stage V or stage IV but with an eGFR projected to decline to <15 mL/min/1.73 m^2^ within 5 y, based on the current eGFR and on the slope of decline calculated by linear regression. All other patients were considered “nonprogressive” (hereafter referred to as “nonprogressives”). CKD stages were defined according to the Kidney Disease: Improving Global Outcome guidelines, with stage IV defined as an eGFR of 15–29 mL/min/1.73 m^2^ and stage V as an eGFR <15 mL/min/1.73 m^2^.^34^

### Assessment of Pretransplant Evaluation Potential by the CKD Clinic’s Medical Team

Progressives were further analyzed according to determination of the pretransplant evaluation potential made by the treating CKD clinic’s medical team, which was classified in the medical charts into the 3 following categories: (1) unsuitable for transplant: patients explicitly deemed unsuitable for transplant by the CKD clinic’s medical team; (2) undetermined pretransplant status: patients who had not been referred for pretransplant evaluation, without a specified reason; and (3) eligible for transplant evaluation: patients considered good potential candidates by the CKD clinic’s medical team, despite potential modifiable limitations (eg, obesity) or those who had initiated the pretransplant workup within the following year.

Referral to the transplant center was not dependent on the result of the pretransplant evaluation. This evaluation was standardized and determined by the transplant center. The decision point for the CKD clinic, on an individual patient basis, was whether or not to conduct this evaluation for the purpose of submitting a consultation request to the transplant center. The transplant center only evaluated patients for whom it received a pretransplant request from the CKD clinics. At our institution, the CKD clinic team bases their referral for transplantation on the Canadian Society of Transplantation consensus guidelines^1^ and on a risk score of mortality for patients older than the age of 70 y.^33^

### Study Approval

This study was evaluated and approved by the Institutional Ethics Committee (project 2024-7335). The reported clinical and research activities are consistent with the Principles of the Declaration of Helsinki. Informed consent was waived by the Ethics Committee given the retrospective nature of the study. The clinical and research activities being reported are consistent with the Principles of the Declaration of Istanbul as outlined in the “Declaration of Istanbul on Organ Trafficking and Transplant Tourism.”

### Statistical Analysis

Comparisons of baseline clinical characteristics were conducted using the *t*-test, Fisher exact test, or chi-square test, as appropriate. Logistic multivariable regressions were used to model the probability of being in the undetermined group. Statistical analyses were performed using Stata version 11.0 (StataCorp, College Station, TX) and SPSS Statistics version 29 (IBM, Armonk, NY). All tests were two-tailed, with a *P* value <0.05 considered statistically significant.

## RESULTS

### Study Population and Comparison Between Nonprogressives and Progressives

**FIGURE 1.**
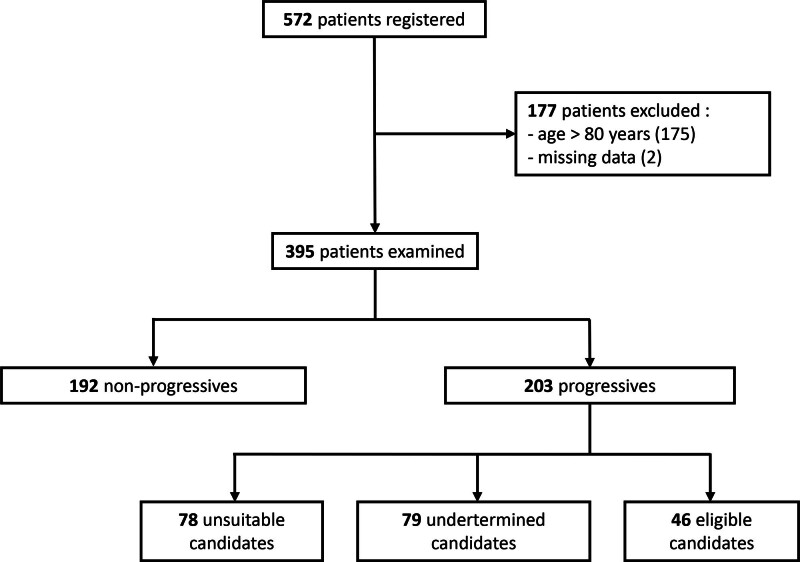
Flow chart.

A total of 572 patients were registered at the CKD clinic as of June 2021. Of these, 177 patients were excluded because they were older than 80 y old (n = 175) or they did not attend their first appointment and were thus registered but not followed at the clinic (n = 2) (Figure 1). Among the remaining 395 patients, 192 were classified as nonprogressives and 203 as progressives. The cohort was primarily male (60%), with a mean age of 68 ± 11 y. The mean eGFR was 20 ± 10 mL/min/1.73 m^2^, and the body mass index was 30 ± 7 kg/m^2^ (Table 1). The majority of patients had hypertension (75%) and diabetes (55%). By design, progressives had a significantly lower eGFR than nonprogressives (13 ± 5 versus 27 ± 9 mL/min/1.73 m^2^, *P* < 0.01). Progressives were also younger (66 ± 12 versus 70 ± 10 y, *P* < 0.01) and had fewer chronic respiratory diseases (21% versus 31%, *P* = 0.03), though other comorbidities were similar. Prognostic risk scores for the 3-y survival did not significantly differ between the 2 groups.

**TABLE 1.** Comparison of progressive versus nonprogressive patients

	All patients (n = 395)	Nonprogressive patients (n = 192)	Progressive patients (n = 203)
Age (y)	68 ± 11	70 ± 10	66 ± 12
Male sex, n (%)	235 (60)	122 (64)	113 (56)
BMI (kg/m^2^)	30 ± 7	30 ± 8	30 ± 7
Hypertension, n (%)	297 (75)	144 (75)	153 (75)
Diabetes, n (%)	216 (55)	105 (55)	111 (55)
Ischemic heart disease, n (%)	112 (28)	58 (30)	54 (27)
Peripheral vascular disease, n (%)	54 (14)	23 (12)	31 (15)
Cerebrovascular disease, n (%)	27 (7)	11 (6)	16 (8)
Heart Failure, n (%)	57 (14)	27 (14)	30 (15)
Dysrythmia, n (%)	64 (16)	30 (16)	34 (17)
Chronic respiratory disease, n (%)	103 (26)	60 (31)	43 (21)
Active malignancy, n (%)	45 (11)	24 (13)	21 (10)
eGFR, (mL/min/1.73m^2^)	20 ± 10	27 ± 9	13 ± 5
>20, n (%)	168 (43)	153 (80)	15 (7)
16–20, n (%)	84 (21)	36 (19)	48 (24)
10–15, n (%)	99 (25)	3(2)	96 (47)
<10, n (%)	44 (11)	0 (0)	44 (22)
CKD stages			
2, n (%)	4 (1)	4 (2.1)	0 (0)
3, n (%)	54 (14)	54 (28.1)	0 (0)
4, n (%)	208 (53)	134 (70)	74 (37)
5, n (%)	129 (33)	0 (0)	129 (64)
3-y survival prognostic score^a^	6.7 ± 4.7	6.6 ± 4.5	6.7 ± 4.9
<7, n (%)	213 (54)	109 (57)	104 (51)
7–9, n (%)	88 (22)	43 (22)	45 (22)
>9, n (%)	94 (24)	40 (21)	54 (27)

Data are expressed as mean ± standard deviation or n (%). eGFR was calculated using the CKD-EPI formula.

a According to Dusseux et al.^33^

BMI, body mass index; CKD, chronic kidney disease; eGFR, estimated glomerular filtration rate.

### Comparison of Unsuitable Versus Eligible Patients

Eligibility for pretransplant evaluation was assessed by the CKD clinic’s medical team during routine visits for all 203 progressive patients. Of these, 78 (38%) were considered unsuitable candidates (Table 2). The primary reasons for unsuitability were comorbidity burden (51/78, 65%) and patient preference for conservative treatment (23/78, 30%). Among the 78 unsuitable patients, 23 had a prognosis score of <7, predicting a 3-y mortality rate of 30% or less.^33^ Reasons for nonreferral in this subgroup included patient refusal or preference for conservative care (9/23), loss of functional autonomy (5/23), severe obesity (BMI >40 kg/m^2^; 4/23), absolute medical transplant contraindications (3/23), and poor compliance (2/23).

**TABLE 2.** Comparison of unsuitable, eligible, and undetermined patients

	Unsuitable (n = 78)	Undetermined (n = 79)	Eligible (n = 46)	*P* ^a^	*P* ^b^
Age (y)	71 ± 10	66 ± 11	58 ± 13	<0.01	<0.01
≤60, n (%)	6 (8)	18 (23)	24 (52)	<0.01	<0.01
61–70, n (%)	22 (28)	21 (27)	13 (28)		
71–75, n (%)	18 (23)	26 (33)	7 (15)		
≥ 76, n (%)	32 (41)	14 (18)	2 (4.3)		
Male sex, n (%)	49 (63)	40 (51)	24 (52)	0.99	0.15
Ethnicity, n (%)				0.11	0.58
White	72 (92)	73 (92)	39 (85)		
Black	0 (0)	0 (0)	2 (4)		
Hispanic	0 (0)	0 (0)	2 (4)		
First Nation	6 (8)	5 (6)	3 (7)		
Middle Eastern	0 (0)	1 (1)	0 (0)		
BMI (kg/m^2^)	30 ± 8	30 ± 8	29 ± 6	0.39	0.62
Hypertension, n (%)	63 (81)	59 (75)	27 (59)	0.07	0.44
Diabetes, n (%)	49 (63)	43 (54)	19 (41)	0.20	0.33
Ischemic heart disease, n (%)	25 (32)	23 (29)	6 (13)	0.05	0.73
Peripheral vascular disease, n (%)	17 (22)	12 (15)	2 (4)	0.08	0.31
Cerebrovascular disease, n (%)	7 (9)	5 (6)	4 (9)	0.72	0.56
Heart Failure, n (%)	17 (22)	11 (14)	2 (4)	0.13	0.22
Dysrhythmias, n (%)	16 (21)	15 (19)	3 (7)	0.07	0.84
Chronic respiratory disease, n (%)	21 (27)	14 (18)	8 (17)	0.99	0.18
Active malignancy, n (%)	13 (17)	7 (9)	1 (2)	0.26	0.16
Total comorbidities per patient	2.9 ± 1.6	2.4 ± 1.6	1.6 ± 1.3	<0.01	0.04
Prognosis score^c^	9.7 ± 5.2	5.9 ± 3.7	3.2 ± 3.0	<0.01	<0.01
<7, n (%)	23 (30)	43 (54)	38 (83)	<0.01	<0.01
7–9, n (%)	13 (17)	26 (33)	6 (13)		
>9, n (%)	42 (54)	10 (13)	2 (4)		
eGFR, (mL/min/1.73 m^2^)	11 ± 4	15 ± 5	12 ± 4	<0.01	<0.01
>20, n (%)	1 (1)	11(14)	3 (7)	<0.01	<0.01
16–20, n (%)	7 (9)	37 (47)	4 (9)		
10–15, n (%)	48 (62)	21 (27)	27 (59)		
<10, n (%)	22 (28)	10 (13)	12 (26)		
CKD stage				<0.01	<0.01
4, n (%)	12 (15)	53 (67)	9 (20)		
5, n (%)	66 (85)	26 (33)	37 (80)		
Residential proximity from transplant center (km)	153 ± 300	125 ± 258	132 ± 269	0.88	0.52
≤150, n (%)	62 (80)	64 (81)	37 (80)	0.73	0.97
151–250, n (%)	1 (1)	1 (1)	0 (0)		
>250, n (%)	15 (19)	14 (18)	9 (20)		

Data are expressed as mean ± standard deviation or n (%). eGFR was calculated using the CKD-EPI formula.

a Comparison between undetermined and eligible patients

b Comparison between undetermined and unsuitable patients

c According to Dusseux et al.^33^

BMI, body mass index; CKD, chronic kidney disease; eGFR, estimated glomerular filtration rate.

In contrast, 46 (23%) patients were deemed eligible for pretransplant evaluation. Compared with unsuitable patients, eligible candidates were younger (58 ± 13 versus 71 ± 10 y, *P* < 0.01), had less comorbidities (1.6 ± 1.3 versus 2.9 ± 1.6, *P* < 0.01), and had significantly better prognostic scores (3.2 ± 3.0 versus 9.7 ± 5.2, *P* < 0.01). Ethnicity and distance from the nephrology center did not differ between the groups. Thus, the data indicated that unsuitability was derived mainly from medically related issues.

### Comparison Between Patients With Undetermined Status and Others

Among the 203 progressives, 79 (39%) had undetermined status for pretransplant evaluation (Table 2). When compared with the eligible group, these patients were older (66 ± 11 versus 58 ± 13 y, *P* < 0.01), had more comorbidities (2.4 ± 1.6 versus 1.6 ± 1.3, *P* < 0.01), and had worse prognostic scores (5.9 ± 3.7 versus 3.2 ± 3.0, *P* < 0.01). They also had a higher eGFR (15 ± 5 versus 12 ± 4 ml/min/1.73 m^2^, *P* < 0.01) and differed in CKD stage distribution, with fewer undetermined patients in stage V (33% versus 80%, *P* < 0.01). Although not statistically significant, the undetermined group trended toward higher rates of hypertension (75% versus 59%), ischemic heart disease (29% versus 13%), peripheral vascular disease (15% versus 4%), heart failure (14% versus 4%), and dysrhythmias (19% versus 7%). Notably, ethnicity, sex, and residential proximity from the hospital did not differ. When compared with the unsuitable group, the undetermined group were younger (66 ± 11 versus 71 ± 10 y, *P* < 0.01), had fewer comorbidities (2.4 ± 1.6 versus 2.9 ± 1.6, *P* = 0.04), and better prognostic scores (5.9 ± 3.7 versus 9.7 ± 5.2, *P* < 0.01). Their eGFR was also higher (15 ± 5 mL/min/1.73 m^2^ versus 11 ± 4 mL/min/1.73 m^2^, *P* < 0.01). Therefore, patients with undetermined status fell between the unsuitable and eligible groups regarding age and comorbidity burden.

**FIGURE 2.**
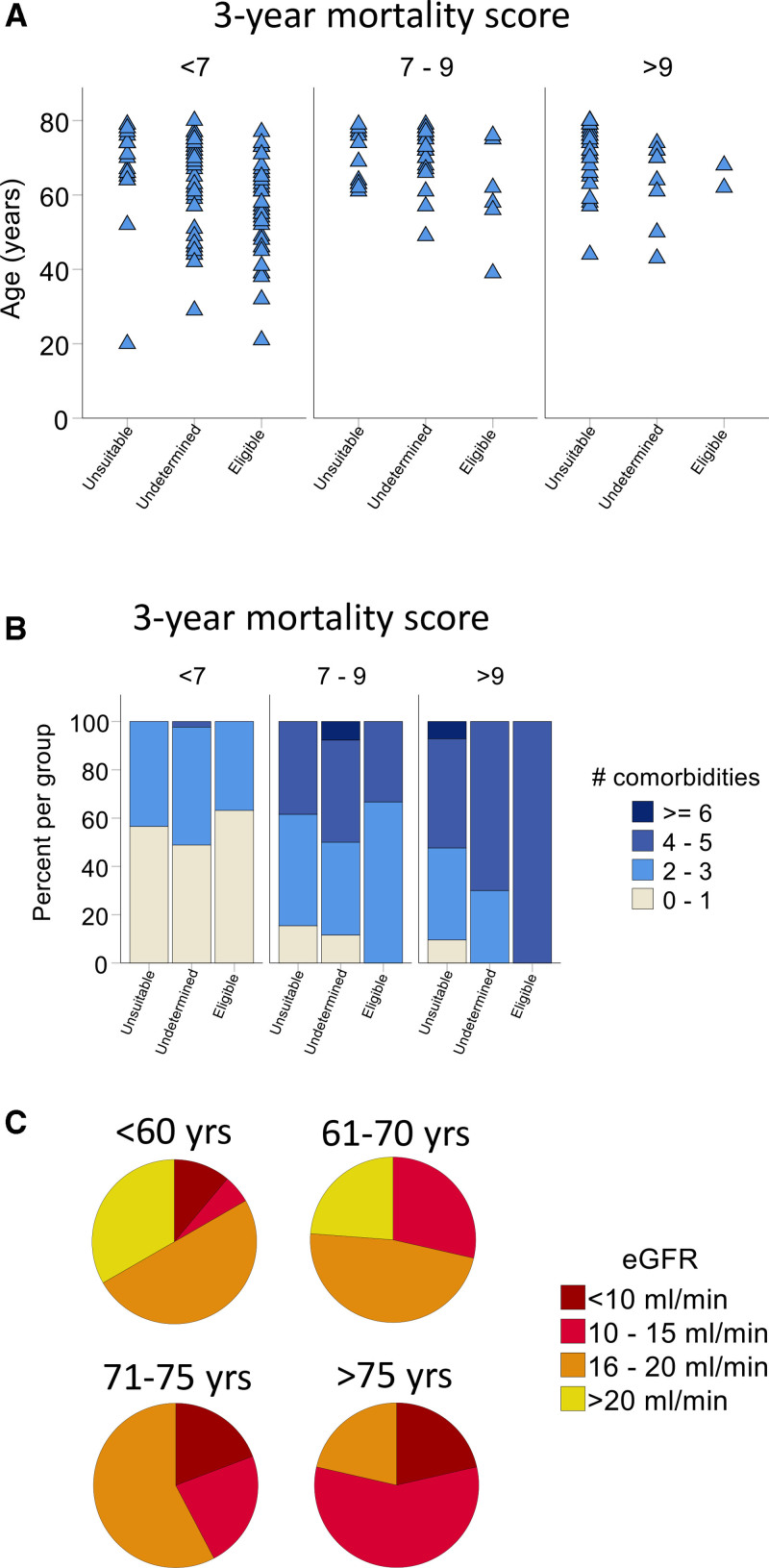
Age, number of comorbidities, eGFR, and survival prognosis. A, For each 3-y survival categories (scores <7, 7–9, and >9), individual patients are displayed as a triangle according to the eligibility status (n = 203). B, For each 3-y survival categories (scores <7, 7–9, and >9), the proportion of patients with 0–1, 2–3, 4–5, and ≥6 comorbidities (among elevated BMI, ischemic heart disease, peripheral vascular diseases, heart failure, dysrhythmia, chronic respiratory disease, cerebrovascular disease, active malignancy, severe behavioral disorder, mobility issues and catheter as dialysis access at dialysis initiation; n = 203). C, Proportion of patients with an eGFR of <10, 10–15, 16–20, and >20 mL/min for each age group. Risk score and definition of comorbidities as described in Dusseux et al.^33^ n = 203.^35^ BMI, body mass index; eGFR, estimated glomerular filtration rate.

Subsequently, we sought to determine whether there was heterogeneity within each group at the individual level. Notably, 43 (54%) of 79 undetermined patients fell within the acceptable prognosis risk score group (score <7) for kidney transplantation. First, we stratified each group by prognosis score and examined the age distribution (Figure 2A). This analysis revealed substantial overlap in the ages of undetermined and eligible patients, particularly among those with an acceptable 3-y mortality risk, estimated at <30% or less (Figure 2A, left). A similar observation was made when comparing the total number of comorbidities between prognosis score subgroups (Figure 2B).

Given these findings, we explored whether a positive relationship existed between age and eGFR among undetermined patients. For instance, it is possible that older, undetermined patients with better eGFR were not considered eligible for transplantation due to the perception that they might become unsuitable candidates by the time they require replacement therapy. Rather, we observed the opposite association (*P* < 0.01; Figure 2C; **Table S1, SDC,** https://links.lww.com/TXD/A801). Specifically, a majority of patients <60 y had an eGFR >16 mL/min/1.73 m^2^, whereas the majority of patients >75 y had an eGFR <15 L/min/1.73 m^2^. Despite the observed differences in age, comorbidities, and eGFR between undetermined and eligible patients, there was considerable overlap, suggesting that patients with similar characteristics have been both considered eligible and undetermined for transplantation.

### Progression Prediction and Sensitivity Analyses

One potential explanation for the nonreferral of potentially eligible patients is that the definition of progression to ESRD used in this analysis may have been inaccurate, leading to an overestimation of the number of progressive patients whose eligibility was not assessed. Therefore, we examined the renal outcomes of all patients—both progressives and nonprogressives—30 mo after the start of follow-up. As shown in Table 3, only 1 (0.5%) of 192 nonprogressives transitioned to RRT, whereas 143 (75%) were still monitored in the CKD clinic, and 10 (5%) were discharged after recovering an eGFR >60 mlLmin/1.73 m^2^. Death occurred in 35 (18%) of 192 patients, with only 1 of these deaths occurring after dialysis initiation. Two patients died from renal causes, both following a serious acute kidney injury without prior CKD progression. Thus, at most, 4 of the 192 nonprogressives could have been misclassified.

**TABLE 3.** Outcome of patients according to progression prediction at 30 mo

	Nonprogressive CKD (n = 192)	Progressive CKD (n = 203)
Transition to RRT	1 (1)	71 (35)
Hemodialysis, n (%)	1 (1)	49 (24)
Peritoneal dialysis, n (%)	0 (0)	13 (6)
Transplantation after dialysis initiation	0 (0)	4 (2)
Preemptive transplantation	0 (0)	5 (3)
CKD with ongoing follow-up clinic, n (%)	143 (75)	82 (40)
Renal recovery, n (%)	10 (5)	0 (0)
Death, n (%)	35 (18)	47 (23)
After dialysis initiation	1 (1)	13 (6)
No dialysis, renal cause	2 (1)	11 (5)
No dialysis, other cause	22 (11)	15 (7)
No dialysis, unknown cause	10 (5)	8 (4)
Loss to follow-up, n (%)	3 (2)	3 (1)

CKD, chronic kidney disease; RRT, renal replacement therapy.

Among the progressive group, 71 (35%) of 203 patients required RRT at 30 mo: 49 patients transitioned to hemodialysis, 13 to peritoneal dialysis, and 4 were transplanted after dialysis initiation, and 5 received preemptive transplantation. Death occurred in 47 (23%) of 203 patients, including 13 deaths after dialysis initiation and 11 deaths from renal causes in patients who opted for conservative management. A significant proportion of progressives (82/203, 40%) continued to be monitored in the CKD clinic without requiring RRT. However, their mean eGFR was 13 mL/min/1.73 m^2^ after 30 mo, with a mean decline of 5 mL/min/y.

Notably, this group includes 22 (11%) patients whose progression has significantly slowed and who do not appear to be heading toward ESRD. Considering that these 22 patients classified as progressive did not exhibit expected progression, we conducted a sensitivity analysis excluding them (**Table S2, SDC,** https://links.lww.com/TXD/A801). Differences in age, total comorbidities, prognostic score, and eGFR were similar to the original analysis when comparing eligible and unsuitable candidates. Additionally, there were no significant differences in individual comorbidities, ethnicity, or residential proximity from the nephrology center. Overall, the results were consistent with the original analysis, with similar inferences drawn from the statistical tests.

We also conducted a second sensitivity analysis in which we excluded the 23 patients who were classified as unsuitable because of their refusal or preference for conservative care (**Table S3, SDC**, https://links.lww.com/TXD/A801). Differences between unsuitable and undetermined patients were similar to the main analysis except for age, which was no longer significantly different. Table 2 and **Table S3, SDC**, https://links.lww.com/TXD/A801 show that 16 of these 23 excluded patients were aged 76 or older.

### Potential Predictors of the Undetermined Status

Finally, we explored the potential characteristics associated with the undetermined status. We conducted multivariable logistic regression analyses including undetermined and eligible patients (n = 125), in which we modeled the probability of being classified as undetermined (Table 4). We first built a model including all the clinical characteristics of Table 2 except the total the number of comorbidities, which was highly collinear with prognosis score. We next built a more parcimonious model, including only the characteristics that showed a difference (at the *P* ≤ 0.15 level) between undetermined and eligible patients. In these models, older age, worse prognosis score, and higher eGFR increased the likelihood of having an undetermined status.

**TABLE 4.** Multivariate predictors of the undetermined status

	OR (95% CI)	*P*	OR^a^ (95% CI)	*P*
Age (y)	1.08 (1.02-1.14)	0.01	1.08 (1.02-1.13)	0.01
Female sex	3.15 (0.94-10.5)	0.06	1.89 (0.69-5.21)	0.22
Non-White ethnicity	0.98 (0.16-6.00)	0.99	0.49 (0.11-2.24)	0.36
BMI (kg/m^2^)	1.05 (0.97-1.15)	0.22		
Hypertension	0.98 (0.24-3.99)	0.98	0.76 (0.22-2.67)	0.67
Diabetes	0.28 (0.07-1.18)	0.08		
Ischemic heart disease	0.30 (0.05-1.93)	0.20	0.34 (0.07-1.83)	0.21
Peripheral vascular disease	0.63 (0.08-5.04)	0.67	0.57 (0.08-3.98)	0.57
Cerebrovascular disease	0.37 (0.06-2.37)	0.30		
Heart failure	0.35 (0.03-3.95)	0.39	0.61 (0.07-5.18)	0.65
Dysrhythmias	1.13 (0.20-6.39)	0.89	1.19 (0.25-5.70)	0.83
Chronic respiratory disease	0.22 (0.04-1.14)	0.07		
Active malignancy	0.92 (0.06-13.6)	0.95		
Prognosis score (1-point)^b^	1.73 (1.23-2.44)	<0.01	1.37 (1.08-1.73)	0.01
eGFR (mL/min/1.73 m2)	1.29 (1.15-1.47)	<0.01	1.26 (1.13-1.41)	<0.01
Distance from transplant center (km)	0.99 (0.99-1.00)	0.44		

Analysis was performed using logistic multivariable regression models, including the 79 patients classified as undetermined and the 46 patients classified as eligible (n = 125).

a Multivariable model including only the characteristics that showed a difference (at the *P* ≤ 0.15 level) between undetermined and eligible patients in Table 2.

b According to Dusseux et al.^33^

BMI, body mass index; CKD, chronic kidney disease; eGFR, estimated glomerular filtration rate.

## DISCUSSION

One of the major findings of this study is the considerable proportion of patients with progressive CKD whose transplant eligibility status was undetermined. This is particularly important because a more thorough assessment of this group could improve pretransplant evaluation referrals. Additionally, it could potentially increase the rate of transplantation, because of additional opportunities for living donation and for the use of otherwise discarded organs from marginal deceased donors. Undetermined patients were generally older, had poorer survival prognostic scores, and slightly higher eGFR than eligible patients. However, we observed a significant overlap at the individual level between undetermined and eligible patients for these characteristics, suggesting that some undetermined patients may, in fact, be suitable candidates for transplantation evaluation.

Previous studies, primarily from the United States, have indicated that race is a major factor limiting transplant referrals. Although Black patients may be referred more often, fewer proceed to pretransplant evaluation, resulting in lower waitlist placement.^13^ In contrast, our CKD clinic population was predominantly White, with few individuals from First Nations or other minority groups, reflective of the local demographics. Unlike earlier reports, ethnicity did not influence referral rates in the current cohort. Similarly, residential proximity from the transplant center did not appear to limit transplant referral. This is consistent with a previous report from the Southeast Kidney Consortium, in which living distance, even >90 miles away, had no statistically significant impact on transplant referrals.^36^

Our results highlight that, although some patients are clearly suitable or unsuitable for kidney transplantation, a large proportion of patients with CKD fall into a gray area. Nephrologists appear more hesitant to refer older patients, especially when eligibility is not obvious, even if the survival prognosis is acceptable. This is particularly true for patients with higher eGFR. Whether this hesitancy is because of concerns about higher eGFR levels delaying the need for transplantation remains unclear. Nonetheless, it is reasonable to assume that within the undetermined group, there are likely a fair proportion of undeclared eligible candidates who could benefit from a transplant evaluation. Delayed or neglected referrals may impact opportunities for preemptive transplantation, living donor identification, and ultimately the chance for successful transplantation.

The 2020 Kidney Disease: Improving Global Outcome guideline on the Evaluation and Management of Candidates for Kidney Transplantation acknowledges the subjectivity in determining transplant suitability and recommend multidisciplinary evaluation to mitigate bias and uncertainty.^37^ To address delays in referral for patients with uncertain eligibility, decisions could be made jointly by the CKD and transplant teams, as is done in some centers. For example, a French center requires transplant team validation of any unfavorable opinion from the CKD nephrologist.^38^ However, in most centers, the transplant team only becomes involved after the standard pretransplant workup is completed, which may disadvantage patients with ambiguous eligibility. To minimize bias, especially ageism, which does not automatically translate to worse outcomes,^39-41^ a Comprehensive Geriatric Assessment by geriatric professionals may help address concerns.^42^

The strengths of this study include its comprehensive analysis of a complete CKD cohort from an academic clinic, representing a large population of >2 million people, reflecting the challenges related to the follow-up of remote patients. Additionally, we validated the definition of CKD progression over time and confirmed that our findings were robust to misclassification. However, this study has limitations. It was conducted at a single center within a publicly funded healthcare system. Data on some socioeconomic factors, such as patient income and employment status, were unavailable. Considering that the Canadian healthcare system is universal, with free medical evaluation, dialysis, transplant and medication, these factors may have less influence here than what has been previously observed in other systems, such as in the United States.^15,16,43^ Patients older than 80 y were excluded; only a minority had a transplant evaluation initiated, limiting the possible analysis for these patients. Of note, although they represent only 0.6% of the kidney recipients aged above 60 y in the United States, they have acceptable survival rates.^29-31^ Finally, referral practices and patient trajectory toward waitlisting are generally guided by local practices, in such a way that some centers may systematically conduct a thorough assessment of all progressive, predialysis patients, whereas others use some sort of selection.

A substantial proportion of patients with progressive CKD have an undetermined transplant eligibility status, with age and subjective comorbidity burden limiting pretransplant evaluation referrals. Moreover, the data suggest that some of these patients are potentially eligible candidates, as indicated by the overlap between characteristics of undetermined and eligible groups. These findings suggest that pretransplant evaluation and preemptive transplantation could be optimized. In the case of predialysis patients, for whom the decision to refer or not to transplant does not seem straightforward for the CKD team, a multidisciplinary approach with earlier involvement of the transplant and geriatric teams could contribute to decision-making. This is particularly true for patients for whom time is a critical factor, especially the elderly.

## ACKNOWLEDGMENTS

The authors wish to thank Mrs Sylvie Dumont and Mrs Sylvie Gendron for their administrative contribution.

## Supplementary Material
