# Supplementary material for: Barriers Limiting Equity in Access to Pretransplant Evaluation Among Predialysis Patients: A Cohort Study
Source: Transplant Direct. 2025 Oct 20;11(11):e1872. doi: 10.1097/TXD.0000000000001872 (PMC12539672; doi:10.1097/TXD.0000000000001872)
Supplement: Supplementary file 1 [file txd-11-e1872-s001.pdf]

SUPPLEMENTAL MATERIAL to Piotte J. *et al.*

- Table S1. Age distribution according to estimated glomerular filtration rates of undetermined patients.
- Table S2. Sensitivity analysis - Comparison of unsuitable, eligible and undetermined patients after exclusion of the 22 patients not progressing at 30 months.
- Table S3. Sensitivity analysis - Comparison of unsuitable, eligible and undetermined patients after exclusion of the 23 patients who elected not to be referred for transplantation.

Table S1. Age distribution according to estimated glomerular filtration rates of undetermined patients (n=79)

eGFR/ age	<60 years	61-70 years	71-75 years	>75 years
>20 ml/min/1.73m^2	6	5	0	0
16-20 ml/min/1.73m^2	9	10	15	3
10-15 ml/min/1.73m^2	1	6	6	8
<15 ml/min/1.73m^2	2	0	5	3

The values indicate the number of patients in each category. $P < 0.01$ by chi-square test

Table S2. Sensitivity analysis - Comparison of unsuitable, eligible and undetermined patients after exclusion of the 22 patients not progressing at 30 months (n=181)

	Unsuitable (n=73)	Undetermined (n=64)	Eligible (n=44)	p-value[1]	p-value[2]
Age (years)	72 ± 10	67 ± 11	58 ± 13	<0.01	<0.01
≤ 60, n (%)	5 (7)	14 (22)	23 (52)		
61-70, n (%)	20 (27)	16 (25)	12 (27)	<0.01	<0.01
71-75, n (%)	18 (25)	22 (34)	7 (16)		
≥ 76, n (%)	30 (41)	12 (19)	2 (5)		
Male sex, n (%)	47 (64)	33 (52)	23 (52)	0.9	0.17
Ethnicity, n (%)				0.14	0.52
Caucasian	67 (92)	59 (92)	37 (84)		
Black	0 (0)	0 (0)	2 (5)		
Hispanic	0 (0)	0 (0)	2 (5)		
First Nation	6 (8)	4 (6)	3 (7)		
Middle Eastern	0 (0)	1 (2)	0 (0)		
BMI, kg/m^2	30 ± 8	29 ± 7	29 ± 6	0.80	0.30
Hypertension, n (%)	60 (82)	49 (77)	26 (59)	0.06	0.53
Diabetes, n (%)	46 (63)	34 (53)	18 (41)	0.24	0.30
Ischemic heart disease, n (%)	24 (33)	19 (30)	6 (14)	0.07	0.72
Peripheral vascular disease, n (%)	16 (22)	11 (17)	2 (5)	0.07	0.53
Cerebrovascular disease, n (%)	6 (8)	5 (8)	4 (9)	0.9	0.9
Heart Failure, n (%)	16 (22)	10 (16)	2 (5)	0.12	0.39
Dysrythmias, n (%)	15 (21)	14 (22)	3 (7)	0.06	1.00
Chronic respiratory disease, n (%)	20 (27)	11 (17)	8 (18)	0.9	0.22
Active malignancy, n (%)	12 (16)	6 (9)	1 (2)	0.24	0.31
Total comorbidities per patient	3.0 ± 1.6	2.5 ± 1.6	1.6 ± 1.3	<0.01	0.10
Prognosis score[3]	9.7 ± 5.2	6.1 ± 3.7	3.2 ± 3.0	<0.01	<0.01
< 7, n (%)	21 (29)	34 (53)	36 (82)		
7-9, n (%)	12 (16)	21 (33)	6 (14)	<0.01	<0.01
> 9, n (%)	40 (55)	9 (14)	2 (4)		
eGFR, (mL/min/1,73m^2)	11 ± 4	15 ± 5	11 ± 4	<0.01	<0.01
> 20 mL/min/1,73m^2, n (%)	1 (1)	5(8)	3 (7)		
16-20 mL/min/1,73m^2, n (%)	5 (7	30 (47)	3 (7)	<0.01	<0.01
10-15 mL/min/1,73m^2, n (%)	46 (63)	19 (30)	26 (59)		
< 10 mL/min/1,73m^2, n (%)	21 (29)	10 (16)	12 (27)		
CKD stage				< 0.01	< 0.01
Stage 4, n (%)	10 (14)	39 (61)	7 (16)		
Stage 5, n (%)	63 (86)	25 (39)	37 (84)		
Prognosis score[3]	9.7 ± 5.2	6.1 ± 3.7	3.2 ± 3.0	<0.01	<0.01
< 7, n (%)	21 (29)	34 (53)	36 (82)		
7-9, n (%)	12 (16)	21 (33)	6 (14)	<0.01	<0.01
> 9, n (%)	40 (55)	9 (14)	2 (4)		
Residential proximity from transplant center (km)	151 ± 303	151 ± 280	118 ± 251	0.53	0.9
≤ 150 km, n (%)	59 (81)	49 (77)	36 (82)		
151-250 km, n (%)	1 (1)	1 (2)	0 (0)	0.62	0.83
> 250 km, n (%)	13 (18)	14 (22)	8 (18)		

Data are expressed as mean ± standard deviation or n (%). eGFR was calculated using the CKD-EPI formula. BMI, Body-mass index; eGFR, estimated glomerular filtration rate; CKD, chronic kidney disease.

[1] Comparison between undetermined and eligible patients

[2] Comparison between undetermined and unsuitable patients

[3] According to Dusseux et al.[33]

Table S3. Sensitivity analysis - Comparison of unsuitable, eligible and undetermined patients after exclusion of the 23 patients who elected not to be referred for transplantation (n=180)

	Unsuitable (n=55)	Undetermined (n=79)	Eligible (n=46)	p-value[1]	p-value[2]
Age (years)	70 ± 10	66 ± 11	58 ± 13	<0.01	0.09
≤ 60, n (%)	6 (11)	18 (23)	24 (52)	<0.01	0.16
61-70, n (%)	18 (33)	21 (27)	13 (28)		
71-75, n (%)	15 (27)	26 (33)	7 (15)		
≥ 76, n (%)	16 (29)	14 (18)	2 (4.3)		
Male sex, n (%)	36 (66)	40 (51)	24 (52)	0.99	0.06
Ethnicity, n (%)				0.11	0.60
Caucasian	50 (91)	73 (92)	39 (85)		
Black	0 (0)	0 (0)	2 (4)		
Hispanic	0 (0)	0 (0)	2 (4)		
First Nation	5(9)	5 (6)	3 (7)		
Middle Eastern	0 (0)	1 (1)	0 (0)		
BMI (kg/m^2)	31 ± 8	30 ± 8	29 ± 6	0.39	0.41
Hypertension, n (%)	43 (78)	59 (75)	27 (59)	0.07	0.68
Diabetes, n (%)	37 (67)	43 (54)	19 (41)	0.20	0.16
Ischemic heart disease, n (%)	19 (35)	23 (29)	6 (13)	0.05	0.57
Peripheral vascular disease, n (%)	13 (24)	12 (15)	2 (4)	0.08	0.26
Cerebrovascular disease, n (%)	7 (13)	5 (6)	4 (9)	0.72	0.23
Heart Failure, n (%)	13 (24)	11 (14)	2 (4)	0.13	0.17
Dysrhythmias, n (%)	11 (20)	15 (19)	3 (7)	0.07	1.00
Chronic respiratory disease, n (%)	18 (33)	14 (18)	8 (17)	0.99	0.06
Active malignancy, n (%)	11 (20)	7 (9)	1 (2)	0.26	0.08
Total comorbidities per patient	3.1 ± 1.6	2.4 ± 1.6	1.6 ± 1.3	<0.01	0.01
Prognosis score[3]	9.8 ± 4.4	5.9 ± 3.7	3.2 ± 3.0	<0.01	<0.01
< 7, n (%)	15 (27)	43 (54)	38 (83)	<0.01	<0.01
7-9, n (%)	9 (16)	26 (33)	6 (13)		
> 9, n (%)	31 (56)	10 (13)	2 (4)		
eGFR, (mL/min/1,73m^2)	11 ± 4	15 ± 5	12 ± 4	<0.01	<0.01
> 20 mL/min/1,73m^2, n (%)	1 (2)	11(14)	3 (7)	<0.01	<0.01
16-20 mL/min/1,73m^2, n (%)	5 (9)	37 (47)	4 (9)		
10-15 mL/min/1,73m^2, n (%)	30 (55)	21 (27)	27 (59)		
< 10 mL/min/1,73m^2, n (%)	19 (34)	10 (13)	12 (26)		
CKD stage				< 0.01	< 0.01
Stage 4, n (%)	8 (15)	53 (67)	9 (20)		
Stage 5, n (%)	47 (85)	26 (33)	37 (80)		
Residential proximity from transplant center (km)	149 ± 305	125 ± 258	132 ± 269	0.88	0.62
≤ 150 km, n (%)	45 (81)	64 (81)	37 (80)	0.73	0.70
151-250 km, n (%)	0 (0)	1 (1)	0 (0)		
> 250 km, n (%)	10 (18)	14 (18)	9 (20)		

Data are expressed as mean ± standard deviation or n (%). eGFR was calculated using the CKD-EPI formula. BMI, Body-mass index; eGFR, estimated glomerular filtration rate; CKD, chronic kidney disease.

[1] Comparison between undetermined and eligible patients

[2] Comparison between undetermined and unsuitable patients

[3] According to Dusseux et al.[33]
